# Supplementary figures and images for: Tracking and perceiving diverse motion signals: Directional biases in human smooth pursuit and perception
Source: PLoS One. 2022 Sep 29;17(9):e0275324. doi: 10.1371/journal.pone.0275324 (PMC9522262; doi:10.1371/journal.pone.0275324)

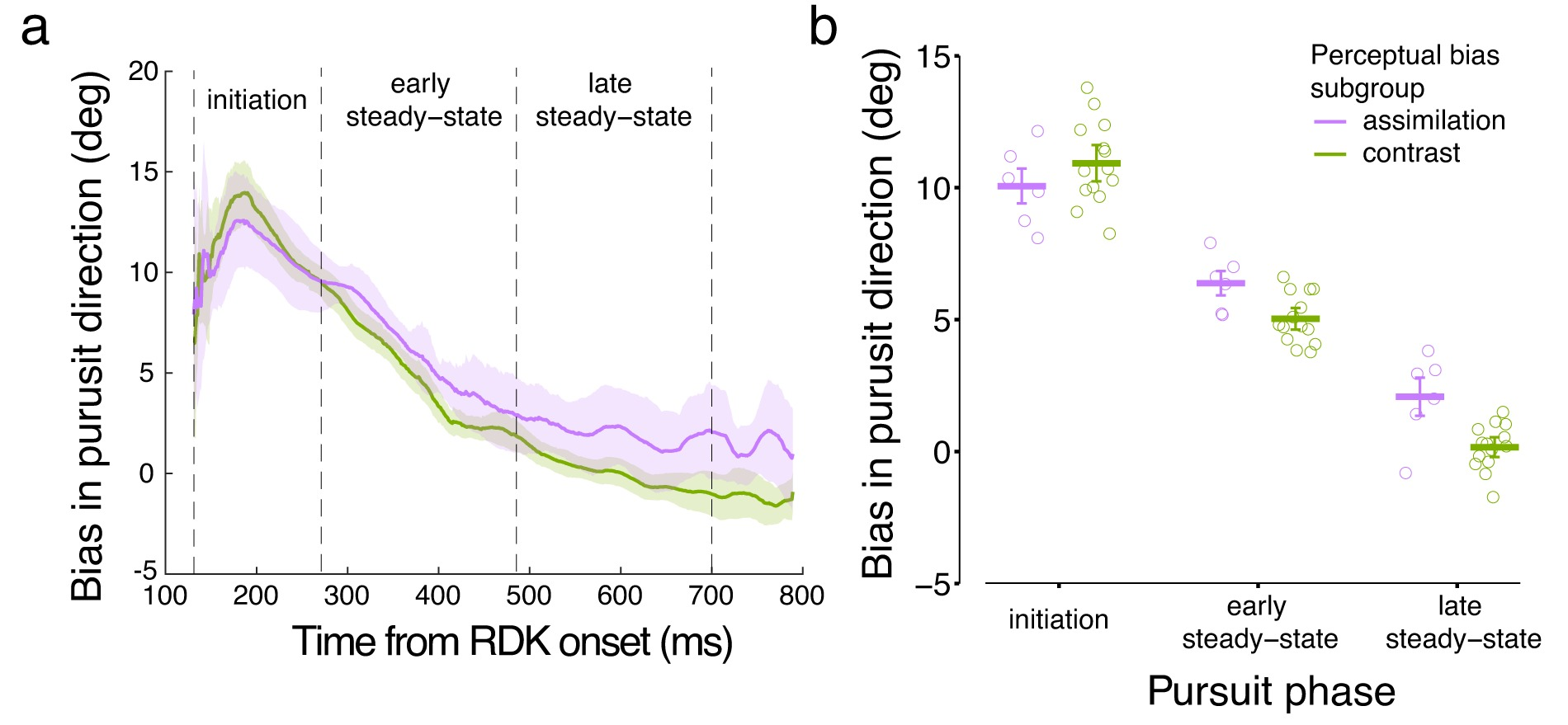

Supplement: S1 Fig — (a) Color indicates the perceptual bias subgroup, see legends in panel (b). Solid lines indicate the mean pursuit bias in each subgroup. Shaded areas indicate the 95% CI. Dashed vertical lines indicate time points of the pursuit onset, and the start, middle point, and end of the steady-state phase analysis window. (b) Biases in pursuit direction in perceptual subgroups across the three pursuit phases. Horizontal bars indicate the mean across observers. Error bars indicate the 95% CI. Circles indicate the mean of individual observers. (TIF) [file pone.0275324.s001.tif]

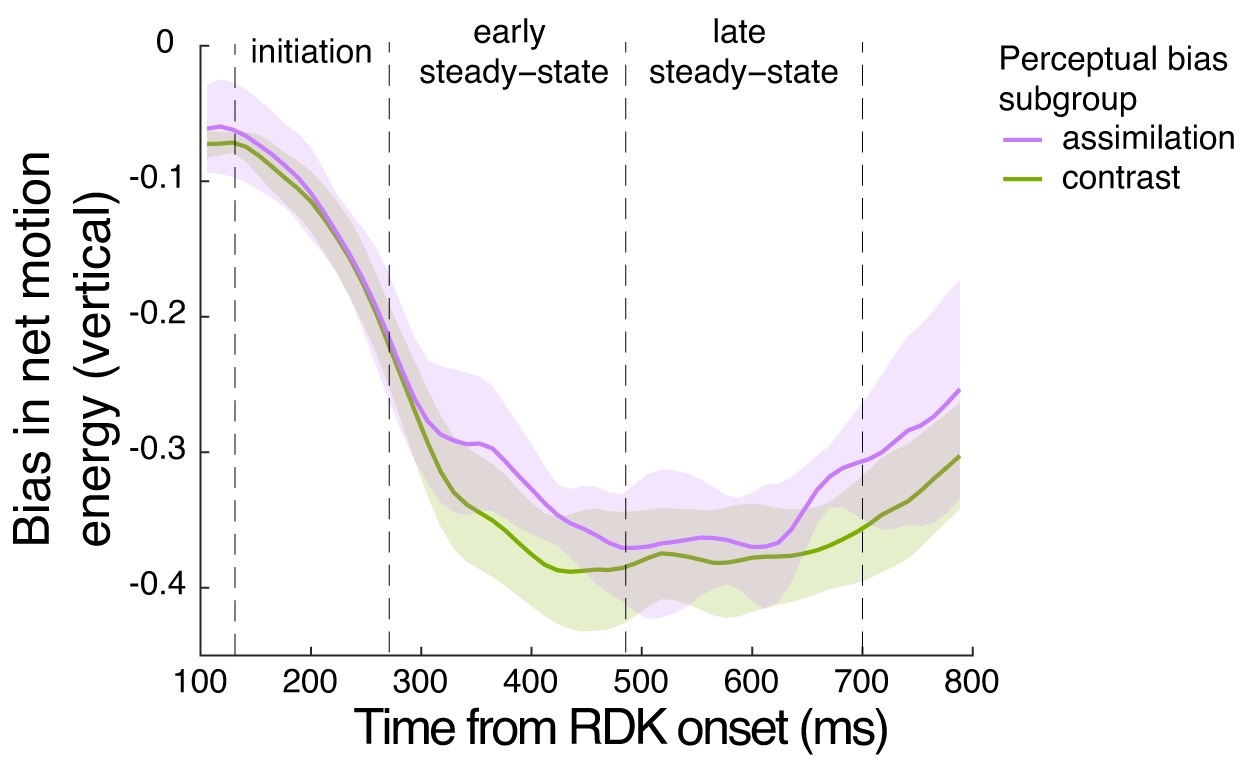

Supplement: S2 Fig — Positive values indicate that there was more motion energy in the same direction as internal motion. Solid lines indicate the mean of each perceptual subgroup. Shaded areas indicate the 95% CI. Dashed vertical lines indicate time points of the pursuit onset, and the start, middle point, and end of the steady-state phase analysis window. (TIF) [file pone.0275324.s002.tif]
