# Supplementary material for: Tracking and perceiving diverse motion signals: Directional biases in human smooth pursuit and perception
Source: PLoS One. 2022 Sep 29;17(9):e0275324. doi: 10.1371/journal.pone.0275324 (PMC9522262; doi:10.1371/journal.pone.0275324)
Supplement: Attachment — Submitted filename: Rebuttal letter.pdf [file pone.0275324.s004.pdf]

We thank the two reviewers and the editor for their constructive comments and positive feedback. All issues raised by the reviewers have been addressed point-by-point as listed below. Changes made to the manuscript are marked in blue ink.

Editor comments:

Thank you for submitting your manuscript to PLOS ONE. After careful consideration, we feel that it has merit but does not fully meet PLOS ONE's publication criteria as it currently stands. Therefore, we invite you to submit a revised version of the manuscript that addresses the points raised during the review process.

The two reviewers found only minor comments to make to your manuscript, as you will see below. Please, read these comments carefully and address them into your revised version. I am fully confident that this next version will answer all the remarks of the reviewers.

Reviewer #1: This work attempted to determine whether pursuit and perception integrate object motion with internal object motion. The authors found that smooth pursuit eye movements relative to object motion were biased in the direction of internal motion, but motion perception was unbiased on average, with striking individual differences between observers. In addition, the perceptual bias was related to the magnitude of the pursuit bias. They interpret that the dissociations between perception and pursuit may reflect the different functional demands of the two systems.

Overall, this is a nice study that is well written and has interesting results. The data are presented properly and support the conclusions. These findings are important and valuable for our understanding of how perception and pursuit are related and interact with each other. I have a few questions/suggestions for improvements to the manuscript (specific comments).

Specific comments:

1. Regarding the perception task, please provide how long it took observers to respond. If there is a time gap between pursuit and perception responses, is it possible that this is related to the difference in bias?

--We now include the average reaction time for the perceptual task in Results (line 330-331). There is indeed a time gap between pursuit and perception responses, already mentioned as a limitation in Discussion, together with potential future directions to address the issue; we have now further clarified this (line 616-630).

2. According to the Methods, the observers were encouraged to focus on the angle of the object's motion trajectory rather than referring to the last RDK position to determine the direction of the object. This is understandable, but I wonder how it can be guaranteed that the observer is not simply using the final position of the RDK as a cue. Please clarify.

--We emphasized in our instructions to observers that using the last RDK position will result in wrong answers because of the spatial jitter. However, this cannot guarantee that the observers strictly followed our instructions (we have rephrased the explanation in Methods, line 189, 193-194). It is difficult to evaluate whether observers used the last RDK position as a reference, because we do not know their "true" and unbiased responses. However, we investigated whether spatial jitter is related to individual differences in perception (see also comment #2 about L193, reviewer #2). Although we randomized spatial jitter, it is possible that observers encountered different randomization of the jitter, leading to different perceptual bias patterns. Therefore, we asked whether the bias caused by spatial jitter was correlated with the perceptual bias, on both across-observer and individual trial-by-trial levels. Specifically, location bias was calculated as the difference between the object motion direction and the direction if pointing the response arrow to the last RDK location. If location bias affected the perceptual bias, we should observe at least a significant across-observer correlation. If observers always followed the last RDK location, we should observe a significant trial-by-trial correlation. We conducted a Pearson's correlation across observers, and a linear mixed effects model [formula: perceptual bias ~ object motion + internal motion + location bias + object motion × location bias + internal motion × location bias + object motion × internal motion + object motion × internal motion × location bias + (1+ location bias | observer)] was fitted on a trial-by-trial basis. We found a significant across-observer correlation (r = -0.48, 95% CI = [-0.76, -0.04], p = 0.03), indicating that observers with larger location biases had smaller perceptual biases. However, this correlation did not seem to explain the different perceptual bias patterns, because for similar location biases, observers showed tendencies for either an assimilation or a contrast perceptual bias (see Fig. R1 below). We did not find significant trial-by-trial correlations, indicated by a non-significant fixed effect of location bias on perceptual bias (estimate = 0.04, 95% CI = [0.01, 0.09], t(27.48) = 1.72, p = 0.10). Together, these results suggested that even if observers were affected by the last RDK location, it did not determine the specific perceptual bias patterns across observers.

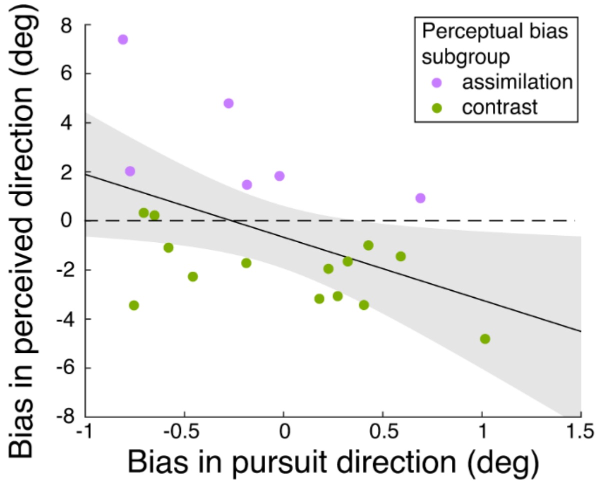

Fig R1. Correlation between the bias in perceived direction and the bias in location across observers (n = 20). Each data point indicates the average value between internal motion conditions for one observer (positive values indicated bias in the direction of internal motion).

Colours indicate the subgroups of perceptual bias. The solid line shows the least squares fit. The shaded area indicates the 95% CI. The dashed line indicates zero perceptual bias.

We prefer not to add these additional analyses to the manuscript, because it does not fully clarify the issue (as the issue could hardly be clarified based on current data), is unlikely to overturn our main finding, and might distract the reader. However, we do agree that it is important to acknowledge this confound, and discussed the potential confounds of the last RDK position in Discussion "Limitations and future directions" (line 591-595).

3. In the Results, the authors describe that "In contrast to the consistent bias in the average motion direction in pursuit, across all observers, perceived direction was not affected by internal motion direction (Fig 6a)." However, since the effect of internal motion on perception varies in each observer, the expression "not affected" is misleading, thus it is recommended to correct the description.

--Here we were referring to the perceptual bias when averaging across all observers, which was not significant. However, we agree that it might be misleading because later we reported the individual difference in perceptual bias, which suggested potential effects of internal motion on perception. We have adjusted the description to "perceived direction across all observers seemed to be not affected" (line 332).

4. It is not clear whether the pattern "a" (assimilation) or "b" (contrast) in figure 6 depends on the influence of attention during the experiment, e.g., attention to internal motion, or attention to global motion, as described in the discussion. So, it would be helpful for readers if the author could further explain what factors determine each pattern (assimilation or contrast).

--We think that attending to different parts of the stimulus might result in individual differences in perceiving the directions, however, the direct correspondence between attention and the specific pattern of bias is unclear, or hard to justify without further evidence, which is beyond the scope of this manuscript. We omitted the discussion about attention (line 603-606) to avoid confusion.

5. The authors described that "Different biases in perception and pursuit might reflect dependency on different types of MT neurons." However, if observers were similarly exposed to visual stimuli, one would assume that MTs would show similar activity because similar retinal slip would occur from RDKs. Nevertheless, please explain additionally how the results of the significant inter-observer individual differences in perception can be understood from the MT activity. It is difficult to understand from the reader's point of view that the difference biases between perception and pursuit could be based on different MT activities.

-- We agree that this discussion does not help to explain the individual difference in perception and have omitted it (line 576-587) to avoid confusion.

<u>Reviewer #2:</u> The current manuscript uses complex stimuli that are moving in the environment, but that also contain internal motion, and asks how human participants make eye movements

(smooth pursuit) to these stimuli, as well as asking about people's subjective experience of them. The authors find that smooth pursuit eye movements are biased in the direction of internal motion, whereas there was more variability in perceptual responses: however, there was still a correlation between perception and pursuit, with participants showing a stronger pursuit bias being more likely to report a directionally aligned perceptual bias. The manuscript is well written and clear, and the analyses seem to be done correctly and interpreted appropriately. The authors have also done an excellent job of making their data and code available, with very comprehensive 'read me' files. I have only a few minor comments that might help to improve the manuscript.

L130 – 'overall link between perception and pursuit' might sound better.

--Rephrased as suggested on line 130.

L193 – I wonder if there is any way to check if some participants did use the last location of the RDK as a reference, and whether this explains any of the variability in perceptual response?

--This comment is related to Reviewer #1 point 2. We have addressed both comments together, as detailed in the response above to Reviewer #1 point 2. To briefly sum up, it is possible that some observers ignored the instruction and used the RDK location as a reference, but it is hard to directly test based on current data whether this occurred. However, we might be able to examine whether the random spatial jitter might have resulted in the individual difference in perception. To evaluate this, we analyzed both across-observer and individual trial-by-trial correlations between the bias caused by spatial jitter (potentially having different patterns for each observer because of the randomization) and the perceptual bias. We found a significant across-observer correlation, but this correlation did not seem to explain the different perceptual bias patterns (see Fig. R1 above). We did not find significant trial-by-trial correlations. Therefore, it is unlikely that the last RDK location determined the specific perceptual bias patterns across observers. We discussed the potential confounds of the last RDK position in Discussion "Limitations and future directions" (line 591-595).

L218 – can you give a ballpark figure for how frequently trials had saccades in them?

--We added the description of the frequency of saccades in Methods "Eye movement recording and analysis" (line 220-221).

L240 – it might be worth being very clear here about what the dependent variables are – I think you are running 2 repeated measures ANOVAs, one with the directional bias for pursuit and the other with the directional bias for perception, but this isn't actually spelled out explicitly.

--The reviewer is correct and we clarified this in Methods (line 243-245).

Fig 3 – having predictions in a graph like this is very helpful, thank you! A very minor point is that the colours don't work very well in black and white (but probably not that important as this is an online only journal).

--We darkened the shade of the blue colour in relevant figures (Fig. 3, 5c, and 6).

L262 – thinking about how you specified this model – is there an argument for including any interactions? I can see why you haven't (as you don't find e.g. a significant interaction between object motion and internal motion in your perceptual rmANOVA) but this seems slightly like hypothesising after the fact given that you included the interactions in those original rmANOVAs. Similarly, Fig 4C/5C seems to suggest that you might expect pursuit bias to interact with object motion direction and internal motion direction, so it might be sensible to include the full set of interactions here.

--We agree that it is possible for pursuit bias to have interactions with object/internal motion. We now include the full set of interactions in the trial-by-trial model (line 266-269), and updated the results (line 377-380). Effects related to pursuit bias remain non-significant.

Fig 7 – is there any way to indicate the confidence interval on this figure?

--We added the confidence interval to Fig. 7 (line 370 for the explanation in the legend). Note that whereas previously we were using the bootstrap method to calculate the confidence interval of the correlation, we now use the Matlab "corrcoef" function (line 363). Both methods/sets of numbers are correct as an estimation. We also updated the related analysis codes in the shared data on OSF.

In addition to the above changes in the main manuscript, in Supporting Information we added the description of how retinal images of the RDK were calculated (line 58) for clarification.
